# Evaluating Molecular Xenomonitoring as a Tool for Lymphatic Filariasis Surveillance in Samoa, 2018–2019

**DOI:** 10.3390/tropicalmed7080203

**Published:** 2022-08-22

**Authors:** Brady McPherson, Helen J. Mayfield, Angus McLure, Katherine Gass, Take Naseri, Robert Thomsen, Steven A. Williams, Nils Pilotte, Therese Kearns, Patricia M. Graves, Colleen L. Lau

**Affiliations:** 1 Australian Defence Force Malaria and Infectious Disease Institute, Enoggera 4051, Australia; 2 School of Public Health, Faculty of Medicine, University of Queensland, Brisbane 4006, Australia; 3 Research School of Population Health, Australian National University, Canberra 2601, Australia; 4 Task Force for Global Health, Decatur, GA 30030, USA; 5 Samoa Ministry of Health, Apia WS 1330, Samoa; 6 Department of Biological Sciences, Smith College, Northampton, MA 01063, USA; 7 Department of Biological Sciences, Quinnipiac University, Hamden, CT 06518, USA; 8 Menzies School of Health Research, Brisbane 4000, Australia; 9 College of Public Health, Medical and Veterinary Sciences, James Cook University, Cairns 4811, Australia

**Keywords:** vector-borne disease, entomology, neglected tropical diseases, elephantiasis, molecular xenomonitoring

## Abstract

Molecular xenomonitoring (MX), the detection of filarial DNA in mosquitoes using molecular methods (PCR), is a potentially useful surveillance strategy for lymphatic filariasis (LF) elimination programs. Delay in filarial antigen (Ag) clearance post-treatment is a limitation of using human surveys to provide an early indicator of the impact of mass drug administration (MDA), and MX may be more useful in this setting. We compared prevalence of infected mosquitoes pre- and post-MDA (2018 and 2019) in 35 primary sampling units (PSUs) in Samoa, and investigated associations between the presence of PCR-positive mosquitoes and Ag-positive humans. We observed a statistically significant decline in estimated mosquito infection prevalence post-MDA at the national level (from 0.9% to 0.3%, OR 0.4) but no change in human Ag prevalence during this time. Ag prevalence in 2019 was higher in randomly selected PSUs where PCR-positive pools were detected (1.4% in ages 5–9; 4.8% in ages ≥10), compared to those where PCR-positive pools were not detected (0.2% in ages 5–9; 3.2% in ages ≥10). Our study provides promising evidence for MX as a complement to human surveys in post-MDA surveillance.

## 1. Introduction

Lymphatic filariasis (LF) is a globally significant parasitic disease which can result in severe morbidity and disability, such as lymphedema, elephantiasis or scrotal hydroceles. Elimination programs for vector-borne diseases such as LF, malaria and onchocerciasis represent significant global health investments. Since 2000, the World Health Organization’s (WHO) Global Programme to Eliminate LF has facilitated mass drug administration (MDA) of anti-filarial drugs to >910 million people in 68 countries [1]. Despite huge successes over >20 years, LF remains endemic in many countries. Most infections are asymptomatic but may still contribute to transmission. Identifying residual infections is therefore critical for programmatic decision-making such as stopping or resuming MDA.

The standard diagnostic test used in LF programmatic surveys detects circulating filarial antigen (Ag), produced by adult worms [2]. Although Ag can be detected quickly and inexpensively in the field using rapid diagnostic tests such as the Alere Filariasis Test Strip [2], they cannot distinguish between current and recently cleared treated infections. Some people remain Ag-positive for months or years after treatment but are not positive for microfilariae (Mf, immature parasites) and do not contribute to ongoing transmission. As countries work towards elimination and LF Ag prevalence drops to very low levels, highly sensitive surveillance methods will be required to detect changes in prevalence and monitor progress. One such alternative indicator of transmission is the presence of Mf in the blood, which confirms active infection. However, Mf may not be detected even if adult worms are present, e.g., if the worms are too young, too old, or have not mated.

Molecular xenomonitoring (MX) involves testing mosquitoes for filarial DNA using the polymerase chain reaction (PCR). Although PCR does not differentiate between a mosquito that is infectious or recently infected, the mosquitoes’ short life span means that the presence of PCR-positive pools indicates that an infectious person was recently nearby [3]. MX may therefore potentially provide a more sensitive measure of where LF transmission is occurring than Ag in humans, as well as an earlier signal of change in transmission. MX has been used in research settings in American Samoa [4], Sri Lanka [5] and India [6], although significant knowledge gaps still exist regarding survey design and interpretation of results. Recent studies have highlighted the need for more efficient mosquito collection methods, better understanding of the relationship between results from MX and human surveys [7], and whether classifying mosquitoes into species groups improves the usefulness of MX results (especially in countries where entomology expertise is limited) [4,8].

LF is endemic in Samoa, transmitted by *Aedes* mosquitoes including the highly efficient *Aedes polynesiensis*, with a flight range of ~150 m [9,10]. Samoa has conducted LF elimination programs under GPELF since 1998 [11], with eight nationwide and two subnational MDAs with DEC and albendazole conducted between 1999 and 2011. Population coverage was sub-optimal in the early campaigns but was over 65% in the five most recent rounds of MDA [11]. Programmatic coverage for the 2018 triple-drug MDA was 83.9% of the eligible population [12]. However, programmatic surveys in 2013 and 2017 [11] and research projects in 2018 [13] showed ongoing transmission. In 2018, Samoa was the first country to distribute nationwide triple-drug MDA (ivermectin, albendazole and diethylcarbamazine) [12]. Evaluating the effectiveness of this intervention is therefore of interest globally, but a key challenge is the ability to detect reductions in human infection prevalence post-MDA. Considering that Ag could persist for months or years after treatment [14], MX might provide a more sensitive surveillance strategy in the post-MDA setting.

In this study, we aimed to evaluate the effectiveness of MX for LF surveillance by comparing the results of spatially representative MX surveys with human Ag prevalence surveys conducted in 2018 (baseline) and 2019 (follow-up) in Samoa. The main objectives were to: (i) estimate the prevalence of LF infection in mosquitoes at baseline and follow-up surveys; (ii) assess the usefulness of MX as an indicator of human Ag prevalence, (iii) compare the usefulness of MX and human Ag for detecting changes in infection prevalence for LF surveillance, and (iv) determine whether separating the mosquitoes into different species prior to aggregating into pooled samples would alter the estimated infection prevalence of the overall sample. Results were considered in a practical context based on what can realistically be achieved on the ground and how findings can be used to assist programmatic decision-making.

## 2. Materials and Methods

### 2.1. Study Region

**Figure 1:**
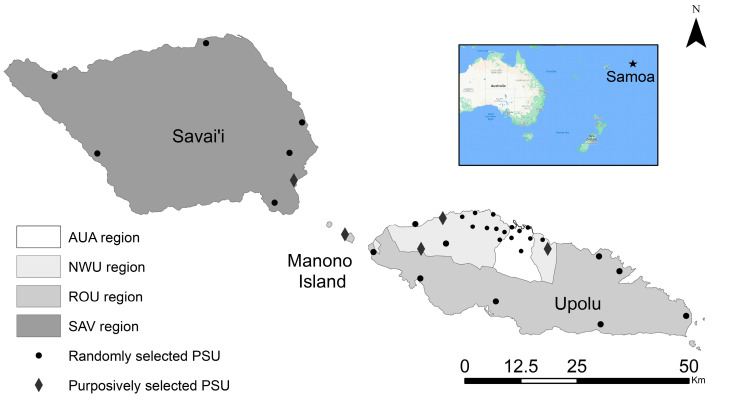
Map of Samoa showing approximate locations of the 35 primary sampling units (PSUs). Villages included in each PSU are given in Supplementary Figure S1.1. Spatial data on country, island, region, and village boundaries in Samoa were obtained from the Pacific Data Hub (pacificdata.org accessed on 8 July 2020) and DIVA-GIS (diva-gis.org, accessed on 12 August 2019). Regions are Apia Urban Area (AUA), North-West Upolu (NWU), Rest of Upolu (ROU) and Savai’i (SAV).

Samoa is a tropical island nation in the South Pacific with approximately 201,316 residents in 2018 [15], the majority of whom live on the islands of Upolu and Savai’i (Figure 1). Villages are predominantly rural, with urbanised areas around the capital of Apia and the Savai’i ferry port of Salelologa. Average rainfall is 3000–6000 mm/year [16] and inland areas remain largely forested. The predominant LF vector species is the day-biting *Ae. polynesiensis*, with evidence that night-biting *Ae. samoanus* (included in *Ae.* (*Finlaya*) subgenus) is likely also able to transmit *Wuchereria bancrofti* [17].

### 2.2. Selection of Primary Sampling Units (PSUs) and Households

Surveys were conducted in Upolu, Savai’i, and Manono islands across 35 PSUs (30 randomly selected and five purposively selected because of high Ag prevalence in previous human surveys). To select the random PSUs for sampling, 30 villages were selected from a line list of 338 villages in the 2016 national census; the first village was randomly selected, and every 11th village from this starting point was included. For eight selected villages with population of <600, the next village on the list was added to the PSU. Five additional villages were purposively selected by the Samoa Ministry of Health due to high Ag prevalence in previous surveys [13]. Therefore, our study included a total of 35 PSUs, each consisting of one or two villages. Average PSU population size was 1355 (range 136 to 4289). The same PSUs were sampled in both years. Human surveys in 2018 and 2019 and the mosquito survey in 2019 included all 35 PSUs. For the 2018 mosquito survey, we were able to sample 28 of the 30 randomly selected PSUs before the start of the MDA, and the five purposively selected PSUs were not included.

Within each PSU, 10 (2018 mosquito survey) or 15 households (2019 mosquito survey; 2018 and 2019 human surveys) were selected using a “virtual walk” method as described previously [13]. If a selected location was not a household, or was uninhabited (destroyed, abandoned, unoccupied), it was replaced by the closest house. If a PSU consisted of two villages, the number of house locations per village was proportionate to each village’s population. Spatial data on country, island, region and village boundaries in Samoa were obtained from the Pacific Data Hub (pacificdata.org; accessed on 8 July 2020) and DIVA-GIS (diva-gis.org; accessed on 9 August 2019). Geographic information systems software ArcMap (v10.6, Environmental Systems Research Institute, Redlands, CA, USA) was used to manage spatial data and produce maps.

### 2.3. Data Collection

#### 2.3.1. Mosquito Survey

**Figure 2:**
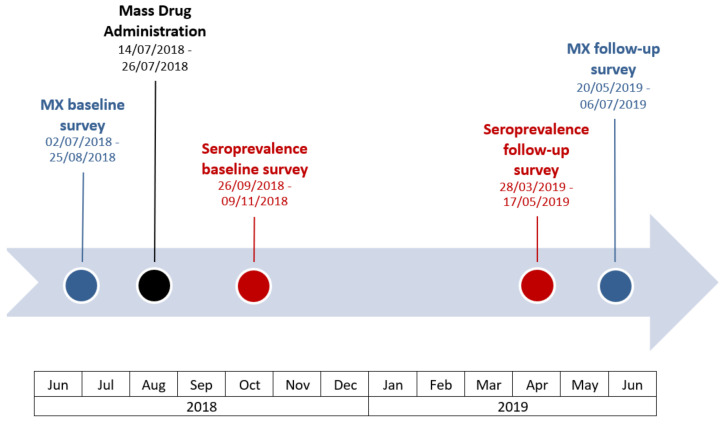
Timeline of 2018 and 2019 surveys (human and mosquitoes) relative to the rollout of the triple-drug mass drug administration [18] in Samoa.

Mosquito surveys were conducted on Upolu and Savai’i from 2 July to 25 August 2018 (within two months pre-MDA), and on Upolu, Savai’i and Manono Island from 20 May to 6 July 2019 (nine to ten months post-MDA). A survey timeline for human and mosquito surveys is provided in Figure 2.

In 2018, the target sample size for mosquitoes was based on a positivity threshold of an upper 1-sided confidence interval <0.25%, using results from Schmaedick et al. (2014) [7] in American Samoa, which found infection prevalence in *Ae. polynesiensis* of >0.25% (0.28%, 95% CI: 0.20%, 0.39%). Using a design effect of two, the estimated target sample size was 13,700 mosquitoes. Based on the work of Chambers et al. (2009) [19] in American Samoa, the expected catch per 10 traps per 24 h was 180 mosquitoes. Thus, for 30 PSUs with 10 trap sites/PSU, the expected catch was 10,800/48 h, the maximum possible time per PSU to enable most PSUs to be surveyed before the start of the MDA. The number of mosquitoes caught in 2018 was lower than expected and therefore, in 2019 the number of households per PSU was increased from 10 to 15 for a total of 525 48 h household trapping periods compared to 280 in 2018.

Traps were placed at each household for 48 h. We used BioGents Sentinel Mosquito Traps (Models 1 and 2 with attractant lures), based on previous findings of their effectiveness when targeting *Aedes* mosquitoes in Samoa [20]. We used a BioGents synthetic lure that mimics the human skin scent, and targets unfed host-seeking mosquitoes. Traps were serviced twice daily in 2018 and once daily in 2019, to collect mosquito bags and replace batteries. Mosquitoes at ambient temperatures can be damaged during transit as they fly around inside bags, hampering species classification. To minimise this, trap-bags of mosquitoes were transported in chilled containers. Mosquitoes were euthanized by placing the trap-bag in a freezer, or in a resealable bag containing an acetone-soaked cotton-ball. Male mosquitoes were discarded.

Female mosquitoes were sorted by species under a stereomicroscope (Make: Olympus, Model: SZ61) using established taxonomic keys [21,22,23]. Four categories were used in 2018: *Ae.* *polynesiensis*, *Ae.* *(Finlaya)* spp. (including *Ae. samoanus*), *Culex* spp., and all other *Aedes* spp. Additional entomological capacity in 2019 enabled sorting into nine categories: *Ae.* *polynesiensis*, *Ae. aegypti*, *Ae. albopictus*, *Ae. upolensis*, *Ae. (Finlaya)* spp., *Aedes* spp. (other), *Cx quinquefasciatus* and *Culex* spp. (other). Mosquitoes that could not be classified at a species level were identified to genus level as *Aedes* spp. (other) or *Culex* spp. (other). All classification at the species level was carried out by qualified entomologists or trained technicians. To reduce workload, initial sorting by sex and genus (*Aedes* vs. *Culex*) was undertaken by non-entomologists who had been trained and were competent in making this initial distinction.

Each species/category of mosquito was placed into pools of 1–25 mosquitoes in 1.5 mL Eppendorf tubes. In 2018, mosquitoes for each species and PSU were pooled separately, but mosquitoes from different households were sometimes combined to minimise the total number of pools per PSU and reduce laboratory costs. Increased catch numbers in 2019 enabled a more precise pooling strategy, where each pool contained a single species from one household. Pooled mosquitoes in open tubes were oven-dried at 60 °C for three hours, and tubes placed in boxes with silica gel desiccant and shipped to Smith College, USA. Samples underwent DNA extraction using the DNeasy Blood and Tissue Kit (Qiagen, Hilden, Germany). Extractions were conducted using a modified version of the manufacturer’s suggested protocol which has been previously described [7]. Following extraction, all samples were tested for the presence of *W. bancrofti* using the real-time PCR assay [24] using all recommended recipes and protocols.

#### 2.3.2. Human Seroprevalence Survey

The baseline survey was conducted from 26 September to 9 November in 2018, within two months after the start of MDA. Considering that Ag persists for at least months after treatment, results were expected to be very similar to pre-MDA Ag prevalence. The follow-up survey was conducted from 28 March to 17 May 2019, seven to nine months post-MDA and immediately prior to the 2019 MX survey (see Figure 2 for survey timeline). The survey design has been previously described [13]. We tested blood samples for Ag using Alere Filariasis Test Strips. For Ag-positive samples, blood slides were prepared and screened for the presence of Mf using standard procedures [13].

### 2.4. Data Analysis

#### 2.4.1. Mosquito Abundance

For each survey year, we calculated mosquito abundance as the number of female mosquitoes collected from all traps over the two-day trapping period. Mosquitoes not classified as either *Culex* or *Aedes* were excluded. We reported abundance by region and species.

#### 2.4.2. Prevalence of PCR-Positive Mosquitoes

Prevalence of mosquitoes infected with *W. bancrofti* was estimated from pool tested results using the R package PoolTestR [25]. The function PoolPrevBayes was used with default uninformative priors to fit Bayesian, mixed effect, multivariable logistic regression models modified for pooled data with variable pool sizes. To determine whether there was an association between mosquito abundance and PCR positivity in the randomly selected PSUs, we used modified logistic regression models adjusting for mosquito genus and clustering at the PSU level, stratified by year. To compare mosquito abundance between the two years, the total number of vectors trapped at each PSU was divided by the number of traps. The presence/absence results for PSUs for *Ae. polynesiensis* and “all species” were mapped to examine the distribution.

##### Mosquito Infection Prevalence in Randomly vs. Purposively Selected PSUs in 2019

To compare prevalence between randomly and purposively selected PSUs in 2019, we fitted a multivariable model with fixed/population effects for region, species and selection method (random/purposive) and random/group effects for PSU. The OR and 95% credible intervals (CrI) for purposively vs. randomly selected villages was used to determine differences and statistical significance. 

To examine the usefulness of MX for detecting changes in infection rates from MDA, we compared the estimated prevalence in 2018 and 2019. For models comparing prevalence between years, we only included data from the 28 PSUs sampled in both years and collapsed species categories to those used in 2018.

#### 2.4.3. Human Antigen Prevalence

Human Ag prevalence for each year was estimated at national and regional levels, using data from the 30 randomly selected PSUs. Sample size calculations were based on numbers required to detect a critical threshold of 2% Ag prevalence in each age group, with a 5% chance of type 1 error, 75% power (when true prevalence is 1%), a design effect of 2.0, and correcting for the finite population. Statistical analyses were performed using Stata (version 16, Stata Corp, College Station, TX, USA). Estimates were calculated using the *proportion* command for each age category (5–9 and ≥10 years), adjusting for selection probability at PSU and individual levels, and for clustering at PSU level. Results were standardized by gender and (for those aged ≥10 years) by age group using 5-year categories in the 2016 census. Finite population correction (FPC) was applied since sampling was without replacement. 

For human Ag, ORs for change in prevalence from 2018 to 2019 were estimated by region and nationally for each age group (5–9 and ≥10 years) using *melogit*, adjusting for selection probability at PSU and individual levels, and for clustering at PSU. Age, gender and region were included as fixed effects.

#### 2.4.4. Comparison of Antigen Prevalence in PSUs with or without PCR-Positive Mosquito Pools

To assess whether the presence of PCR-positive mosquitoes was a useful indicator of infections in humans, we compared Ag prevalence between PSUs with PCR-positive mosquito pools and PSUs where no PCR-positive pools were detected. This analysis was conducted using the randomly selected PSUs where both MX and human Ag surveys were conducted (28 PSUs in 2018, and 30 PSUs in 2019).

#### 2.4.5. Comparison of Human Ag and Mosquito Infection Prevalence between Baseline (2018) and Follow-Up (2019) 

To examine changes over time at the national or regional level, we fitted multivariable models with fixed/population effects for vector species and, random/group effects for each PSU at baseline (2018), and random/group effects for the temporal change in each PSU. Region was not included as a covariate as approximate leave-one-out cross validation [26] indicated including it did not improve the model. To examine changes over time by region, we modified the model to include fixed/population effects for each region in 2018 and fixed/population effects for the temporal change. From these models we calculated ORs (which approximate prevalence ratios as prevalence was low) and 95% CrI to quantify differences between years at national, regional and PSU levels. As a sensitivity analysis, we fitted alternative models adjusting for genus rather than species, or without adjustment for either. To compare each surveillance method in the baseline and follow-up surveys, we used prevalence estimates by year in 28 PSUs that were surveyed in both years. We calculated ORs (2019 vs. 2018) to determine significant changes in prevalence between years.

## 3. Results

### 3.1. Mosquito Abundance

In 2018, a total of 8506 female mosquitoes were trapped (Table 1), with a mean of 320 per PSU (range 147–781, median 278), equating to a mean of 30.4 mosquitoes per trap over 280 traps. *Culex* were the predominant category (n = 4333, 48%), followed by *Aedes polynesiensis* (n = 2498, 28%), other *Aedes* spp. (n = 1443, 16%) and *Ae.* (*Finlaya*) spp. (n = 698, 8%). The predominant species in the *Culex* and *Aedes* (other) categories were *Cx. quinquefasciatus* and *Ae. aegypti*, respectively. Numbers of *Ae. polynesiensis* by PSU ranged from 9 to 248 (mean 89, median 69).

In 2019 a total of 34,299 female mosquitoes were trapped (Table 2), with a mean of 980 per PSU (range 290–3719, median 855), equating to an average of 65.3 mosquitoes per trap over 525 traps. *Cx. quinquefasciatus* was the predominant species (*n* = 17,807, 52%), followed by *Ae. polynesiensis* (*n* = 10,540, 31%), *Ae. aegypti.* (*n* = 3396, 10%) and *Ae.* (*Finlaya*) spp. (*n* = 1377, 4%). Numbers of *Ae. polynesiensis* varied by PSU with a range of 22 to 1046 (mean 301, median 173). Abundance for all species by PSU for both years is available in Supplementary Tables S2.1 and S2.2.

### 3.2. Precence and Prevalence of PCR-Positive Mosquitoes

**Figure 3:**
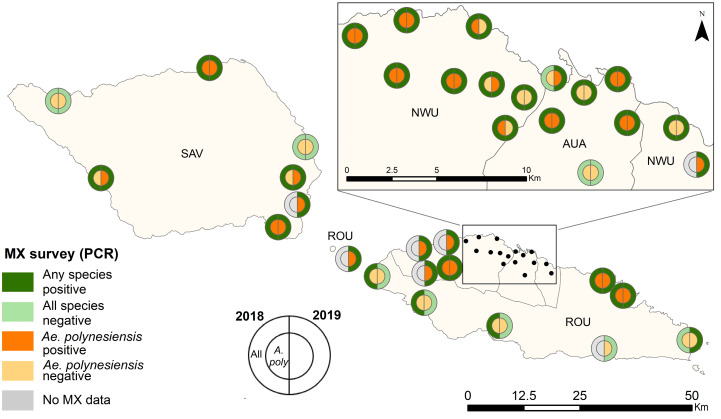
Presence of female mosquitoes (*Ae. polynesiensis* and “any species”) PCR-positive for *W. bancrofti* by primary sampling unit (PSU), Samoa. Data from 2018 shown in the left semicircle, and 2019 in the right semicircle.

In 2018, 50% (14/28) of PSUs returned at least one PCR-positive pool of *Ae. polynesiensis* and 82% (23/28) returned at least one PCR-positive pool of “any species”. Results were similar in 2019, with 63% (22/35) of PSUs returning at least one PCR-positive pool of *Ae. polynesiensis* and 80% (28/35) returning at least one PCR-positive pool of “any species” (Figure 3).

**Figure 4:**
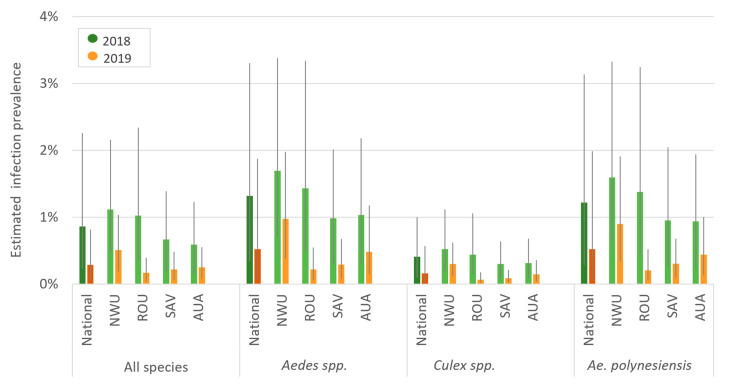
Estimated infection prevalence (%) by mosquito species, region and year (using data from the 28 randomly selected primary sampling units (PSUs) surveyed in both 2018 and 2019), Samoa. AUA = Apia Urban Area; NWU = North-West Upolu; ROU = Rest of Upolu; SAV = Savai’i. Values provided in Supplementary Table S3.1.

In 2018, mosquitoes were sorted into 475 pools (mean 18 mosquitoes/pool, range 1–25); 18% (86/475) of pools were positive for W. bancrofti DNA by PCR. The highest positivity rate of pools was 30% in *Aedes* spp. (other) (31/105 pools), followed by 24% for Ae. polynesiensis (33/136 pools) (Supplementary Table S3.1). Nationally, estimated infection prevalence in all species combined was 0.9% (95% credible interval [CrI] 0.2–2.3), with the highest prevalence in *Aedes* spp. (other) (1.6%) followed by *Ae. polynesiensis* (1.2%). Highest prevalence was found in the regions of Northwest Upolu (NWU) followed by Rest of Upolu (ROU) (Figure 4, Supplementary Table S3.2). At the sub-regional level, NWU (Musumusu and Faleapuna) and southeast Savai’i (Papa and Tafua) had the highest prevalence (Supplementary Table S3.3).

In 2019, mosquitoes were sorted into 2638 pools (mean 13 mosquitoes/pool, range 1-25). The highest positivity rate of pools within the randomly selected PSUs was 9.2% (56/612 pools) in *Ae. polynesiensis* followed by 6.5% for Ae. aegypti (70). Most pools consisted of *Cx. quinquefasciatus* (39%) or *Ae. polynesiensis* (27%). Within all PSUs, 7% (172/2638) of pools were PCR-positive. The highest positivity rate of pools was 14% in *Ae. polynesiensis* (102/723), followed by 7% for *Ae. aegypti* (33/469). Estimated infection prevalence in all species combined was 0.3% (95% CrI 0.05–1.0%), with the highest prevalence in *Ae. polynesiensis* (0.6%) (Figure 4). Highest prevalence was again observed in NWU followed by Apia Urban Area (AUA) (Figure 4). At the PSU level, three purposively chosen PSUs in NWU not sampled in 2018 Lauli’i (7.5%), Fasito’o Tai (7.1%) and Faleasiu (6.4%)—had the highest prevalence (Supplementary Figure S3.1, Supplementary Table S3.4).

Combining results of the 28 PSUs that were sampled in both years, estimated mosquito infection prevalence for the country was lower in 2019 than 2018 for both the entire sample (“all species”) (0.3% vs. 0.9%) and *Ae. polynesiensis* (0.5% vs. 1.2%). This decreasing trend was observed across all species categories nationally and in all regions, although not all differences were statistically significant (Figure 4). There was no association between PSU mosquito abundance and PCR positivity in 2018 (OR 0.84, 95% CrI: 0.53–1.32) or 2019 (OR 0.85, 95% CrI: 0.71–1.01). Mosquito abundance was therefore not adjusted for in other analyses.

#### Mosquito Infection Prevalence in Randomly vs. Purposively Selected PSUs in 2019

In 2019, crude infection prevalence in all mosquito species in randomly selected PSUs was 0.3% (95% CrI 0.05–1.05%), lower than the 1.1% (95% CrI 0.1–3.9%) in purposively selected PSUs (Supplementary Table S3.1). This difference remained statistically significant after adjusting for mosquito species, region, and clustering within PSUs (OR 0.24, 95% CrI 0.06–0.86). For both randomly and purposively selected PSUs, the highest prevalence was observed in *Ae. polynesiensis* (0.6% and 1.8%, respectively), followed by *Ae. aegypti* (0.5% and 1.5%, respectively).

### 3.3. Human Antigen Prevalence

**Figure 5:**
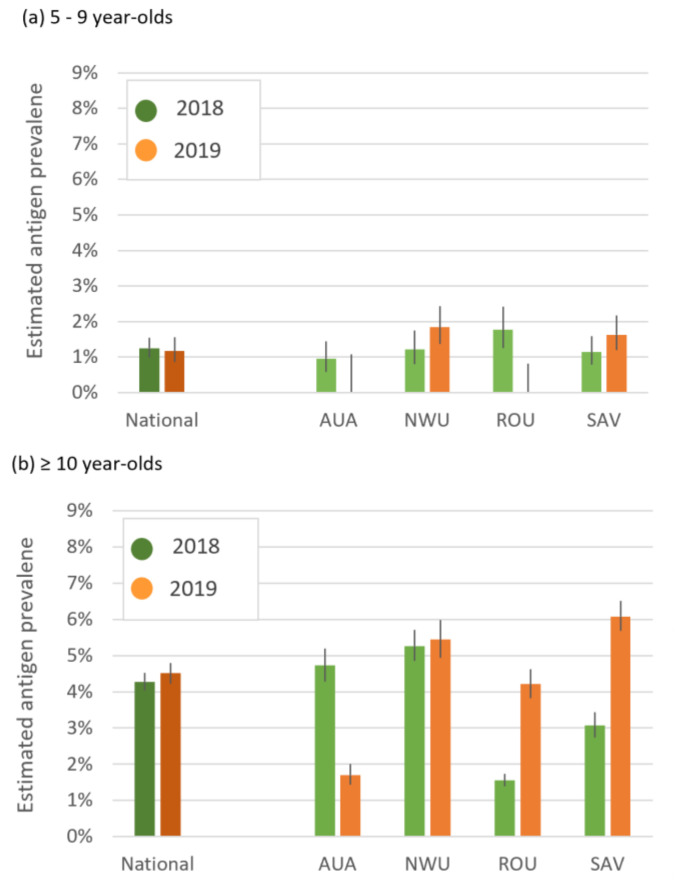
Adjusted antigen prevalence from human survey in 2018 and 2019 for 30 randomly selected primary sampling units (PSUs) in Samoa for (**a**) 5–9 year-olds and (**b**) ≥10 year-olds. Adjusted for selection probability at PSU and individual levels, clustering at the PSU level, finite population correction, and standardized for age and gender.

In the 2018 baseline survey, we recruited 1542 participants aged 5–9 years and 1551 participants aged ≥10 years from 30 randomly selected PSUs [13]. In the 2019 follow-up survey, we recruited 1811 participants aged 5–9 years and 1815 participants aged ≥10 years from the same 30 PSUs. Participants numbers in 2018 and 2019 in each of the four regions are given in Supplementary Tables S4.1 and S4.2. Figure 5 shows that in both 2018 and 2019, the adjusted Ag prevalence at national and regional levels were higher for the ≥10 age group. After stratification by region, ORs for the 5–9-year-old group could not be estimated in two regions (AUA and ROU) because there were no Ag-positives in 2019.

### 3.4. Comparison of Antigen Prevalence in PSUs with or without PCR-Positive Mosquito Pools

**Figure 6:**
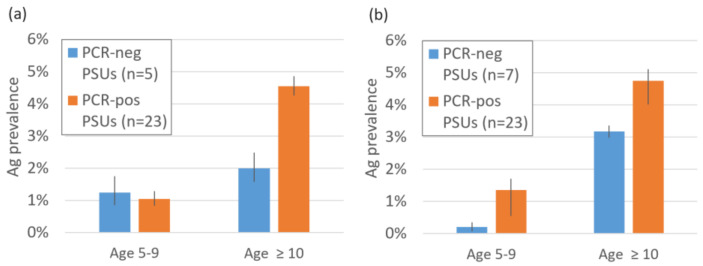
Estimated Ag prevalence in primary sampling units (PSUs) with and without PCR-positive mosquito pools in (**a**) 2018 and (**b**) 2019.

In 2018, of the 28 random PSUs where both MX and human Ag surveys were conducted in both years, PCR-positive pools were found in 82% of PSUs (23/28). This decreased to 77% of PSUs in 2019 (23/30). Ag prevalence in PCR-positive PSUs was higher than in PCR-negative PSUs for both age groups and years except for in 5–9-year-olds in 2018 (Figure 6). The distribution of PCR-positive and Ag-positive PSUs is given in Supplementary Figure S5.1.

### 3.5. Comparison of Human Ag and Mosquito Infection Prevalence between Baseline (2018) and Follow-Up (2019) Surveys

**Figure 7:**
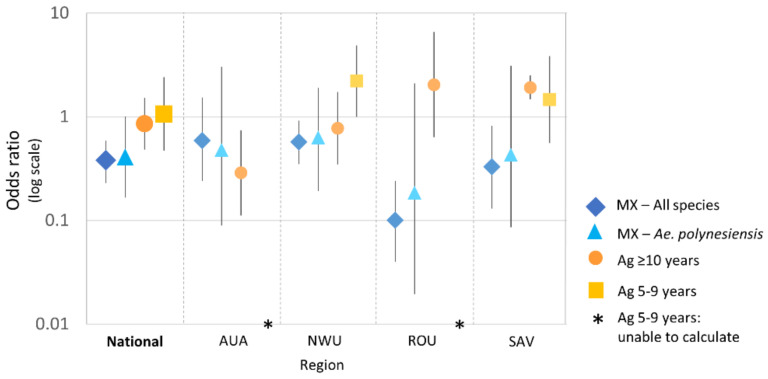
Change in prevalence from 2018 to 2019 in Samoa, expressed as an odds ratio (OR), for mosquito infection prevalence (MX for all species and *Ae. polynesiensis*), and human antigen prevalence (in those aged ≥10 years, and 5–9 years). Given the low prevalence, the ORs are approximately equal to prevalence ratios. ORs < 1 indicate decrease in infection prevalence in 2019 compared to 2018, ORs > 1 indicate an increase, and OR of 1 indicate no change. OR could not be calculated for 5–9-year-olds in AUA and ROU because no antigen-positive cases were detected in these groups in 2019. AUA = Apia Urban Area; NWU = North-West Upolu; ROU = Rest of Upolu; SAV = Savai’i.

MX showed a significant reduction in mosquito infection prevalence from 2018 to 2019 nationally and in all regions for “all species” and *Ae. polynesiensis* (Figure 7). After adjusting for mosquito species and clustering within PSUs, infection prevalence at the national level was lower in 2019 than 2018 (OR 0.4, 95% CrI 0.2–0.6) and in three regions (NWU: OR 0.6 [95% CrI 0.4–0.9], ROU: OR 0.1 [95% CrI 0–0.2], SAV: OR 0.3 [95% CrI 0.1–0.8]). Estimated prevalence declined in AUA, but the difference was not statistically significant (OR 0.6, 95% CrI 0.2–1.5). Given the low prevalence, these ORs closely approximate prevalence ratios. For example, infection prevalence in mosquitoes in ROU was ~90% (i.e., 1–0.1) lower in 2019 than in 2018.

There was no significant change in Ag prevalence between the two surveys in participants aged ≥10 years or 5–9 years at the national level. Variable patterns were observed at the region level, with a significant increase in Ag prevalence between 2018 and 2019 in 5–9-year-olds in NWU (OR 2.2, 95% CI 1.0–4.9) and in ≥10-year-olds in SAV (OR 1.9, 95% CI 1.5–2.5) but a significant decrease in ≥10-year-olds in AUA (OR 0.3, CI 0.1–0.7).

Even though our study was designed to detect differences at national and regional levels, we also observed statistically significant reductions in mosquito infection prevalence between 2018 and 2019 in 10 PSUs, and prevalence did not increase in any PSUs (Supplementary Table S6.1). Adjustment for genus or species had little effect on the point estimate or confidence intervals for the difference in prevalence between 2018 and 2019 at national, regional or PSU levels (Supplementary Figures S7.1 and S7.2)

## 4. Discussion

This study demonstrated the value of MX as an effective tool for LF surveillance, with a potentially higher sensitivity than human Ag prevalence for detecting early changes in infection levels following triple-drug MDA. After one round of triple-drug MDA in Samoa, MX detected a decline in prevalence of infected mosquitoes, while there was no significant change in Ag prevalence between the two human community surveys conducted six months apart. We compared MX results for the main vector *Ae. polynesiensis* and all species combined and found that temporal trends in mosquito infection prevalence were similar between regions.

Higher numbers of female mosquitoes were caught in 2019 than 2018, partly due to drier conditions in 2018 and partly due to the additional seven PSUs and larger number of households per PSU (15 instead of 10) surveyed in 2019. In 2019, survey teams were also more proactive with trap placements to increase catches (e.g., if few mosquitoes were trapped in the first 24 h, the trap was moved to another part of the house for the next 24 h). The increased catch numbers in 2019 resulted in more pools, leading to higher workload and laboratory costs but provided more precise infection prevalence estimates. Although the number of PCR-negative PSUs is potentially related to mosquito abundance (i.e., the more mosquitoes tested, the higher the probability that one of them will test positive), analysis confirmed that prevalence of infection in mosquitoes was not correlated with the average catch numbers per trap. It is, however, possible that the number of PCR-positive PSUs in 2018 could be an underestimate due to the low catch numbers caused by a lower survey effort (ten traps per PSU than 15) and adverse weather conditions during and prior to trapping.

Several factors could potentially influence the estimated mosquito infection prevalence. These include prevalence of infectious humans nearby, mosquito biting rate, and the chance that infected and uninfected mosquitoes are trapped. We believe that a decrease in the prevalence of infectious humans nearby is the most likely explanation for a reduction in mosquito infection prevalence in Samoa. Although mosquito behavioural changes or seasonal changes are theoretical possibilities that could increase or decrease transmission intensity, there is no evidence that these factors are operating over the time scale studied. If MX is to be used as a routine surveillance strategy, future studies should be designed to specifically answer this question and optimise interpretation of MX results.

Mosquitoes categorised as “*Aedes* other” in 2018, most likely to be *Ae. aegypti,* had a higher pool PCR positivity rate and infection prevalence than *Ae. polynesiensis* in 2018, but the observation was reversed in 2019. *Aedes* spp. had a dramatically higher pool positivity rate and infection prevalence than that of *Culex* spp. The large numbers of *Culex* spp. pools therefore diluted the overall infection prevalence of “all species” combined. Where prior knowledge of endemic mosquito species distribution and infection prevalence are available, this can be used to inform the pooling strategy for MX as to whether certain mosquito categories can be excluded from PCR analysis.

Comparison between mosquito categories showed higher infection prevalence in *Aedes* spp. than *Culex* spp. Nevertheless, analyses using “all species” or only *Ae. polynesiensis* had similar spatial and temporal trends, though the larger sample sizes with “all species” resulted in tighter confidence intervals, illustrating one advantage of using all mosquito samples available. Sorting mosquitoes by species requires substantial expertise. MX surveys could be greatly simplified if sorting were restricted to the genus level. Promisingly, despite the differences in infection prevalence between *Culex* and *Aedes* mosquitoes and between *Ae. polynesiensis* and other *Aedes* mosquitoes, adjustment for genus or species had little effect on the point estimate or confidence interval for the difference in prevalence between 2018 and 2019 at national, regional or PSU levels. This finding suggests that, for the purposes of detecting temporal trends, sorting by genus or species in our study made little difference to the results or their interpretation. Whether this observation can be generalised to future surveys in Samoa or elsewhere requires further investigation. If the relative abundance of species captured were substantially different between two time points or two survey locations to be compared, failing to sort by species may bias comparisons.

The pool PCR positivity rate and mosquito infection prevalence were three times greater in the purposively selected than the randomly selected PSUs. This was expected since the purposively selected PSUs had historically high LF prevalence, which highlights the importance of local knowledge for identifying areas of high transmission. Mosquito infection prevalence in randomly selected PSUs was lower in 2019 than 2018 nationally and in each region (Figure 4 and Figure 7), but this difference was not statistically significant for AUA.

When comparing MX and Ag for post-MDA surveillance, we found a statistically significant decrease in infection prevalence in mosquitoes at the country level for “all species” and *Ae. polynesiensis*, but no significant change overall in Ag prevalence in 5–9-year-olds or in those aged ≥10 years. This could be due to a slower change in Ag compared to mosquito infection prevalence. These results demonstrate each surveillance method’s sensitivity to changing prevalence, and when used together may provide a more complete picture. MX may provide a more accurate picture of the transmission potential from humans to mosquitoes at specific time points, whereas Ag, due to its more gradual decline, provides a smoother trend over time. Although the significant reduction in mosquito infection prevalence suggests an encouraging impact on LF transmission by the MDA, transmission from humans to mosquitoes was not completely interrupted, suggesting the possibility of ongoing transmission. Pedersen et al. (2009) [3] used modelling and empirical data to estimate thresholds of 0.085% L3 larvae prevalence in mosquitoes or 0.65% for any stage of filarial worm infection in mosquitoes; but these predicted thresholds have not been validated with field studies [3].

Our results should be considered in light of the study’s limitations. In 2018, the sample size of households required to trap sufficient mosquitoes was calculated using the best available evidence, but dry weather conditions led to lower-than-expected catches. Operational challenges in 2018 also led to simplified pooling by reducing the number of mosquito categories. In 2019, protocol changes and wetter conditions led to increased catches. Mosquitoes were pooled between households in 2018, so prevalence estimates could not be adjusted for household-level clustering. Comparisons of relative magnitude and changes in human and mosquito infection prevalence over time depend on close overlap and frequent contact between the two populations. In most cases, the traps were located at or near the households selected for the human survey, except where this was not possible (no suitable location, residents not home for second survey etc.), in which case they were placed as close as possible (for example at a neighbouring property). We do not believe this is a major limitation as even if placed at the same households, the traps would be sampling mosquitoes that may have acquired infection at other nearby households.

In conclusion, our study suggests that in the immediate post-MDA period, MX might be more sensitive than Ag for detecting changes in transmission. However, if the goal is to detect the presence of residual transmission, Ag and MX surveys may provide complementary information. In MX, using both the primary mosquito species and other species increased the sample size and improved the ability to detect transmission and changes in prevalence. In our study, adjusting for species made very little difference to estimates of infection prevalence, suggesting that the labour-intensive process of sorting mosquitoes into species categories did not influence the overall results or their interpretation. In countries where the expertise required to trap and identify mosquitoes is limited, omitting the requirement to classify them into species groups could make MX more feasible. Further research is required to determine whether our findings are generalisable to future surveys in Samoa and to other settings.

## Figures and Tables

**Table 1:** Number of female mosquitoes caught by species category and region, Samoa 2018. Regions are Apia Urban Area (AUA), North-West Upolu (NWU), Rest of Upolu (ROU) and Savai’i (SAV).

Region	*Ae. polynesiensis*	*Ae. (Finlaya)* spp.	*Aedes* spp. (Other)	*Culex* spp. (All)	All Species
AUA	467	242	179	848	1736
NWU	1140	60	803	1954	3957
ROU	313	178	296	847	1634
SAV	578	218	165	684	1645
**Total**	**2498**	**698**	**1443**	**4333**	**8972**

**Table 2:** Number of female mosquitoes caught by species category and region, Samoa 2019. Regions are Apia Urban Area (AUA), North-West Upolu (NWU), Rest of Upolu (ROU) and Savai’i (SAV).

Region	*Ae. polynesiensis*	*Ae. aegypti*	*Ae. (Finlaya)* spp.	*Aedes* spp. (Other)	*Cx. quinquefasciatus*	*Culex* spp. (Other)	Other	All Species
AUA	1232	623	15	54	2965	0	2	4891
NWU	3443	1432	107	283	5823	0	9	11,097
ROU	2642	822	206	508	7179	1	3	11,361
SAV	3223	519	1049	291	1840	23	5	6950
**Total**	**10,540**	**3396**	**1377**	**1136**	**17,807**	**24**	**19**	**34,299**

## Data Availability

All relevant data are within the paper. We are unable to provide individual-level antigen prevalence data and demographic data because of the potential for breaching participant confidentiality. The communities in Samoa are very small, and individual-level data such as age, sex, and village of residence could potentially be used to identify specific persons. For researchers who meet the criteria for access to confidential data, the data are available on request from the Human Ethics Officer at the Australian National University Human Research Ethics Committee, email: human.ethics.officer@anu.edu.au.

## Supplementary Materials

The following supporting information can be downloaded at: https://www.mdpi.com/article/10.3390/tropicalmed7080203/s1, Figure S1.1: Regions and approximate locations of the selected primary sampling units; Table S2.1: Number of female mosquitoes caught in 2018; Table S2.2: Number of female mosquitoes caught in 2019; Figure S2.1: Number of female mosquitoes caught in (a) 2018 and (b) 2019 in Samoa; Table S3.1: Estimated prevalence of female mosquitoes PCR-positive for *W. bancrofti* by region and species category; Figure S3.1: Estimated prevalence of female mosquitoes infected with *W.bancrofti* by primary sampling unit (PSU) and species category; Table S3.2: Estimated prevalence of PCR-positive female mosquitoes for W. bancrofti by region and species category; Table S3.3: Estimated prevalence of female mosquitoes PCR-positive for *W. bancrofti* in 2018; Table S3.4 Estimated prevalence of female mosquitoes PCR-positive for *W. bancrofti* in 2019; Table S4.1: Number of participants in each of the four regions of Samoa in the 28 randomly selected primary sampling units included in 2018 and 2019 human and mosquito surveys; Table S4.2: Number of participants in each of the four regions of Samoa in the 30 randomly selected primary sampling units included in 2018 and 2019 human surveys; Figure S5.1: Distribution of PCR-positive mosquitoes and Ag-positive humans by primary sampling unit; Table S6.1: Odds ratios for change in prevalence of female mosquitoes PCR-positive for *W.bancrofti*, between 2018 and 2019; Figure S7.1: Change in mosquito infection prevalence by region from 2018 to 2019; Figure S7.2: Change in mosquito infection prevalence by primary sampling unit (PSU) from 2018 to 2019.
