# Supplementary material for: Evaluating Molecular Xenomonitoring as a Tool for Lymphatic Filariasis Surveillance in Samoa, 2018–2019
Source: Trop Med Infect Dis. 2022 Aug 22;7(8):203. doi: 10.3390/tropicalmed7080203 (PMC9414188; doi:10.3390/tropicalmed7080203)
Supplement: Supplementary file 1 [file tropicalmed-07-00203-s001.zip › Tropicalmed-1846014 - supplementary updated-1.pdf]

Tropical Medicine and Infectious Disease

Article

Evaluating Molecular Xenomonitoring as a Tool for Lymphatic Filariasis Surveillance in Samoa, 2018-2019

Brady McPherson [1,*,†], Helen J. Mayfield [2,†], Angus McLure [3], Katherine Gass [4], Take Naseri [5], Robert Thomsen [5], Steven A. Williams [6], Nils Pilotte [7], Therese Kearns [8], Patricia M. Graves [9] and Colleen L. Lau [2]

[1] Australian Defence Force Malaria and Infectious Disease Institute, Enoggera 4051, Australia;
[2] School of Public Health, Faculty of Medicine, University of Queensland, Brisbane 4006, Australia;
[3] Research School of Population Health, Australian National University, Canberra 2601, Australia;
[4] Task Force for Global Health, Decatur, GA 30030, USA;
[5] Samoa Ministry of Health, Apia, Samoa;
[6] Department of Biological Sciences, Smith College, Northampton, MA 01063, USA;
[7] Department of Biological Sciences, Quinnipiac University, Hamden, CT 06518, USA;
[8] Menzies School of Health Research, Brisbane 4000,
[9] College of Public Health, Medical and Veterinary Sciences, James Cook University, Cairns,4811 Australia;
* Correspondence: brady.mcpherson@defence.gov.au
† These authors contributed equally to this work.

Supplementary material

1. Map of Primary Sampling Units

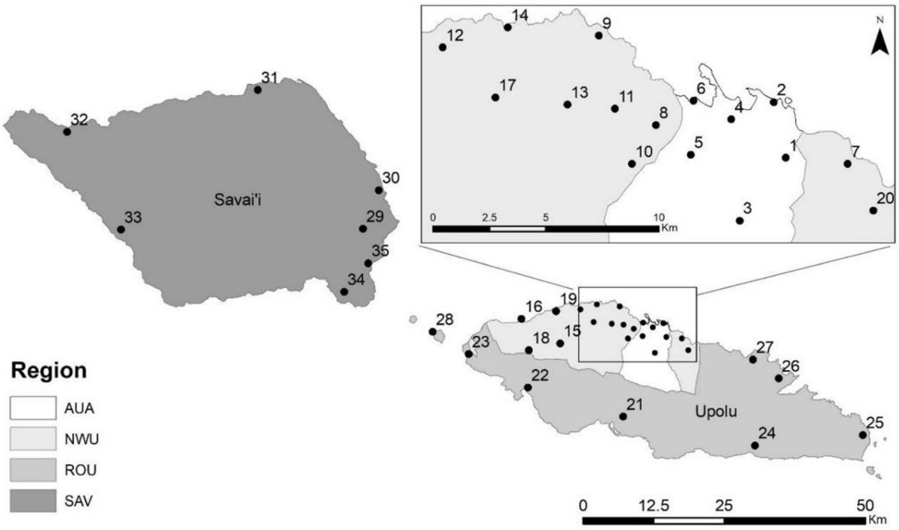

Region	PSU	Village(s)
AUA	1	Vaivase Tai
	2	Vaiala Tai + Vaiala Uta
	3	Avele + Letava
	4	Vaimea + Fugalei
	5	Vaimoso
	6	Vaitoloa
NWU	7	Letogo
	8	Vaiusu
	9	Puipaa
	10	Ulułoloa
	11	Vaitele Fou
	12	Lotosoa
	13	Nuu
	14	Tuanai
	15	Fasitoo Uta
	16	Vailuutai
	17	Leauvaa
	18	Fasitoo Tai †
	19	Faleasiu †
	20	Laulii †

Region	PSU	Village(s)
ROU	21	Fusi
	22	Faleseela
	23	Manono Uta
	24	Salani + Utulaelae
	25	Mutiatele + Saleaaumua
	26	Falefa
	27	Musumusu + Faleapuna
	28	Salua †
SAV	29	Lalomalava + Safua
	30	Lano
	31	Safotu
	32	Sataua
	33	Sagone
	34	Papa + Tafua
	35	Salelologa †

† denotes purposively selected PSU

Figure S1. 1 Regions and approximate locations of the selected primary sampling units (PSUs), Samoa. Regions are Apia Urban Area (AUA), North-West Upolu (NWU), Rest of Upolu (ROU) and Savai'i (SAV).

2. Mosquito Abundance.

Table S2. 1 Number of female mosquitoes caught in 2018 by species category and primary sampling unit.

PSU No.	Village(s)	*Ae. polynesiensis*	*Ae. (Finlaya)* spp.	*Aedes* spp. (other)	*Culex* spp. (all)	All species
1	Vaivase Tai	63	3	55	108	229
2	Vaiala Tai + Vaiala Uta	178	91	0	121	390
3	Avele + Letava	151	60	8	128	347
4	Vaimea + Fugalei	10	87	0	174	271
5	Vaimoso	12	0	35	160	207
6	Vaitoloa	53	1	81	157	292
7	Letego	248	11	38	134	431
8	Vaiusu	36	1	73	87	197
9	Puipaa	25	3	37	156	221
10	Ulualoloa	31	3	91	208	333
11	Vaitele Fou	9	0	35	135	179
12	Lotosoa	245	6	63	282	596
13	Nuu	75	13	55	638	781
14	Tuanai	177	10	161	201	549
15	Fasitoo Uta	142	5	221	52	420
17	Leauvaa	152	8	29	61	250
21	Fusi	11	11	76	114	212
22	Faleseela	39	9	33	348	429
23	Manono Uta	39	3	109	96	247
25	Mutiatele + Saleaaumua	107	113	4	87	311
26	Falefa	51	6	73	97	227
27	Musumusu + Faleapuna	66	36	1	105	208
29	Lalomalava + Safua	111	61	11	257	440
30	Lano	71	40	26	147	284
31	Safotu	159	17	42	86	304
32	Sataua	73	5	27	42	147
33	Sagone	57	29	56	127	269
34	Papa + Tafua	107	66	3	25	201
**Total**		**2498**	**698**	**1443**	**4333**	**8972**
**Mean per PSU**		**89**	**25**	**52**	**155**	**320**

Table S2. 2. Number of female mosquitoes caught in 2019 by species category and primary sampling unit.

PSU No.	Village	*Ae. polynesiensis*	*Ae. aegypti*	*Ae. albopictus*	*Ae. upolensis*	*Ae. (Finlaya)* spp.	*Aedes spp.* (other)	*Cx. quinquefasciatus*	*Culex spp.* (other)	Other	All species
1	Vaivase Tai	362	161	0	0	1	12	327	0	1	864
2	Vaiala Tai + Vaiala Uta	567	61	0	0	0	6	343	0	0	977
3	Avele + Letava	96	74	0	0	12	10	596	0	0	788
4	Vaimea + Fugalei	61	114	0	0	0	14	573	0	0	762
5	Vaimoso	83	95	0	0	0	7	814	0	0	999
6	Vaitoloa	63	118	0	0	2	5	312	0	1	501
7	Letego	469	74	0	0	31	38	1035	0	1	1648
8	Vaiusu	22	34	0	0	4	0	230	0	0	290
9	Puipaa	50	34	0	0	1	0	217	0	0	302
10	Ululoloa	85	71	0	0	0	12	898	0	2	1068
11	Vaitele Fou	85	158	0	0	1	8	336	0	1	589
12	Lotosoa	959	131	0	0	11	50	231	0	0	1382
13	Nuu	81	247	0	0	1	19	994	0	0	1342
14	Tuanai	164	149	0	0	1	28	314	0	1	657
15	Fasitoo Uta	109	75	0	0	6	21	205	0	0	416
16	Vailuutai	173	87	0	0	2	12	221	0	0	495
17	Leauvaa	132	94	0	0	4	8	250	0	0	488
18	Fasitoo Tai*	413	140	0	0	7	49	246	0	0	855
19	Faleasiu*	446	77	0	0	4	21	332	0	3	883
20	Laulii*	255	61	0	0	34	17	314	0	1	682
21	Fusi	116	154	0	0	31	2	775	0	0	1078
22	Faleseela	69	49	0	0	17	18	1644	0	0	1797
23	Manono Uta	62	76	0	0	5	13	786	0	0	942
24	Salani + Utulaelae	1046	133	0	0	102	180	2257	0	1	3719
25	Mutiatele + Saleaaumua	683	131	0	0	32	268	928	0	1	2043
26	Falefa	150	71	0	0	10	4	457	0	0	692
27	Musumusu + Faleapuna	156	76	0	0	4	2	247	1	1	487
28	Salua*	360	132	0	0	5	21	85	0	0	603
29	Lalomalava + Safua	558	196	0	0	207	77	221	0	0	1259
30	Lano	330	9	0	0	252	23	204	3	3	824
31	Safotu	385	72	0	0	14	12	251	1	1	736
32	Sataua	387	49	0	0	68	4	289	0	1	798
33	Sagone	479	61	0	0	229	12	161	0	0	942
34	Papa + Tafua	855	7	0	0	184	137	332	18	0	1533
35	Salelologa*	229	125	0	0	95	26	382	1	0	858
**Total**		**10540**	**3396**	**0**	**0**	**1377**	**1136**	**17807**	**24**	**19**	**34299**
**Mean per PSU**		**301**	**97**	**0**	**0**	**39**	**32**	**509**	**0.7**	**0.5**	**980**

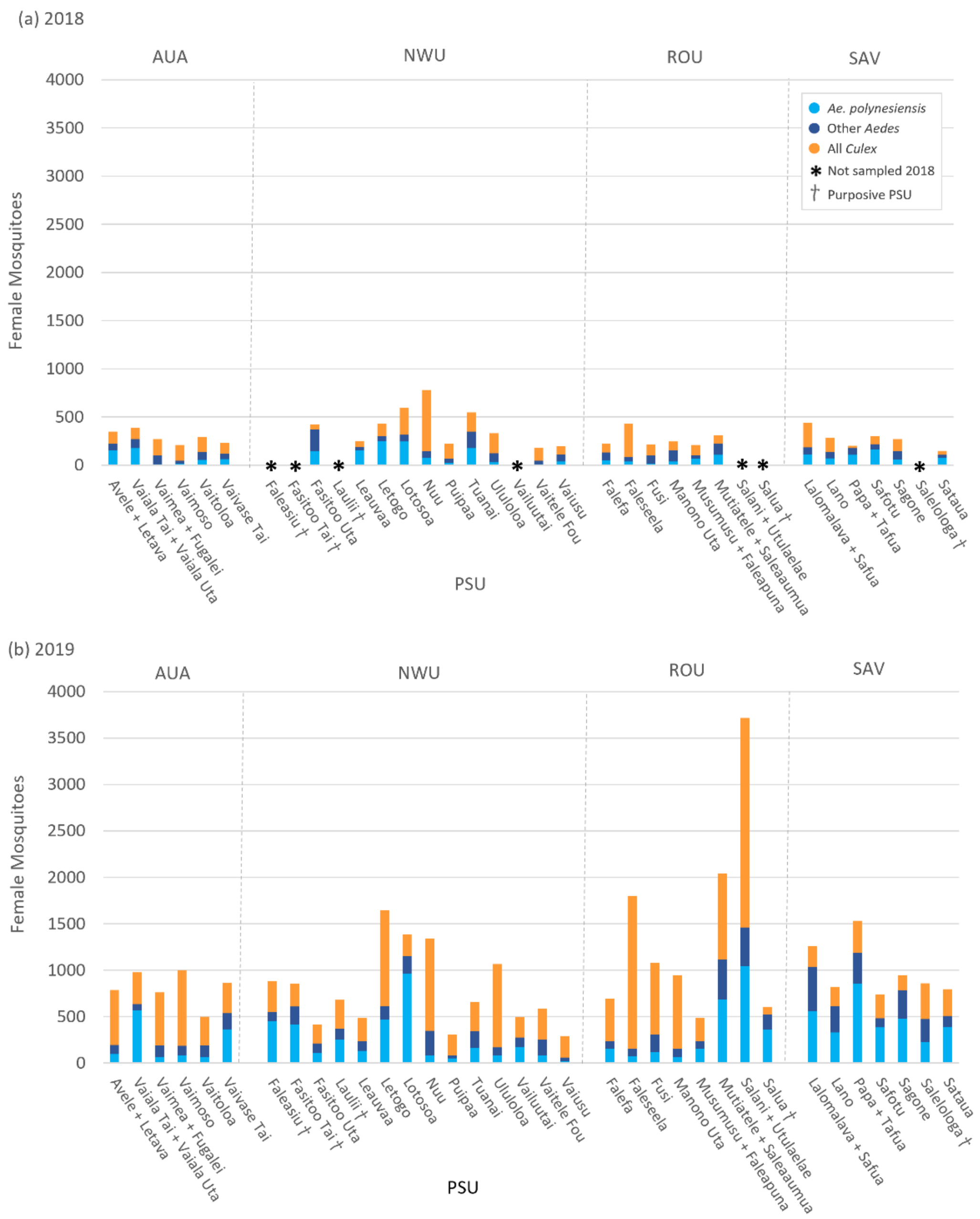

Figure S2. 1. Number of female mosquitoes caught in (**a**) 2018 and (**b**) 2019 in Samoa. Sorted by species category and primary sampling unit. Sampling efforts were increased from ten traps per primary sampling unit (PSU) in 2018 to 15 traps per PSU in 2019.

3. Estimated Prevalence of PCR-positive Mosquitoes, by Species

Prevalence of mosquitoes infected with *Wuchereria. bancrofti* was estimated from pool tested results using the R package PoolTestR[23]. When estimating prevalence for a single PSU for a single species, genus, or without any adjustment for mosquito species, the function PoolPrev was used to calculate the maximum likelihood prevalence. The function PoolPrevBayes was used with default uninformative priors to fit Bayesian, mixed effect, and multivariable logistic regression models modified for pooled data with variable pool sizes.

Table S3. 1. Detection of *W. bancrofti* DNA in female mosquito pools in 2018 and 2019 in Samoa. Results for 2019 (35 primary sampling units - PSUs) are spilt by randomly (R) or purposively (P) selected PSUs. Purposive PSUs were not surveyed in 2018 (28 PSUs).

Year	Species category	Females (n)		Pools (n)		Positive Pools (n)		Positive pool (%)		Estimated infection prevalence* (%) (95% Crl)	
		R	P	R	P	R	P	R	P	R	P
2018	*Ae. polynesiensis*	2451	NA	136	NA	33	NA	24.3	NA	1.2 (0.3-2.9)	NA
	Ae. (Finlaya) spp.	219	NA	33	NA	1	NA	3.0	NA	0.4 (0-1.6)	NA
	Aedes spp. (other, including *aegypti)*	1866	NA	105	NA	31	NA	29.5	NA	1.6 (0.4-4.1)	NA
	Culex spp. (all)	3970	NA	201	NA	21	NA	10	NA	0.4 (0.1-1.0)	NA
	All species	8506	NA	475	NA	86	NA	18.1	NA	0.9 (0.2-2.3)	NA
2019	*Ae. polynesiensis*	8,833	1,703	612	111	56	46	9.2	41.4	0.6 (0.1-2.4)	1.8 (0.2-6.9)
	Ae. (Finlaya) spp.	1,232	145	170	28	1	1	0.6	3.6	0.2 (0-0.8)	0.5 (0-2.6)
	Ae. aegypti	2,861	535	399	70	26	7	6.5	10	0.5 (0.1-2)	1.5 (0.1-5.6)
	Aedes spp. (all others)	1,002	134	169	36	4	3	2.4	8.3	0.5 (0-1.7)	1.8 (0.1-4.9)
	Cx. quinquefasciatus	16,448	1,359	917	100	25	3	2.7	3	0.1 (0-0.4)	0.3 (0-1.2)
	Culex spp. (other)	23	1	8	1	0	0	0	0	0 (0-0)	0-0 (0-1)
	Unidentified	15	4	14	3	0	0	0	0	0 (0-0)	0 (0-0)
	All species	30,414	3,881	2,289	349	112	60	4.9	17.2	0.3 (0.05-1.0)	1.1 (0.1-3.9)

Table S3. 2. Estimated prevalence of PCR-positive female mosquitoes for *W. bancrofti* by region and species category in 2018 and 2019 in Samoa. CI = confidence interval, AUA=Apia Urban Area; NWU = North West Upolu; ROU = Rest of Upolu; SAV = Savai'i.

	Region	2018 Prevalence (%) (95% CI)	2019 Prevalence (%) (95% CI)
All species	National	0.86 (0.63,1.40)	0.29 (0.23,0.53)
	NWU	1.11 (0.65, 1.04)	0.51 (0.32,0.53)
	ROU	1.03 (0.68,1.32)	0.17 (0.13,0.23)
	SAV	0.66 (0.48,0.73)	0.22 (0.15,0.26)
	AUA	0.59 (0.44,0.64)	0.25 (0.17,0.30)
All *Aedes*	National	1.32 (0.97,1.99)	0.52 (0.46,1.35)
	NWU	1.70 (0.99,1.68)	0.98 (0.59,1.00)
	ROU	1.43 (0.95,1.91)	0.22 (0.16,0.33)
	SAV	0.98 (0.69,1.03)	0.29 (0.21,0.39)
	AUA	1.03 (0.73,1.15)	0.48 (0.34,0.70)
All *Culex*	National	0.41 (0.31,0.59)	0.16 (0.14,0.41)
	NWU	0.52 (0.32,0.59)	0.30 (0.19,0.32)
	ROU	0.44 (0.29,0.62)	0.07 (0.05,0.11)
	SAV	0.30 (0.21,0.34)	0.09 (0.06,0.13)
	AUA	0.31 (0.22,0.36)	0.15 (0.10,0.21)
Ae. polynesiensis	National	1.22 (0.94,1.92)	0.52 (0.47,1.46)
	NWU	1.60 (0.95,1.73)	0.90 (0.56,1.01)
	ROU	1.38 (0.94,1.87)	0.21 (0.16,0.31)
	SAV	0.95 (0.69,1.09)	0.31 (0.22,0.37)
	AUA	0.94 (0.67,1.00)	0.44 (0.31,0.56)

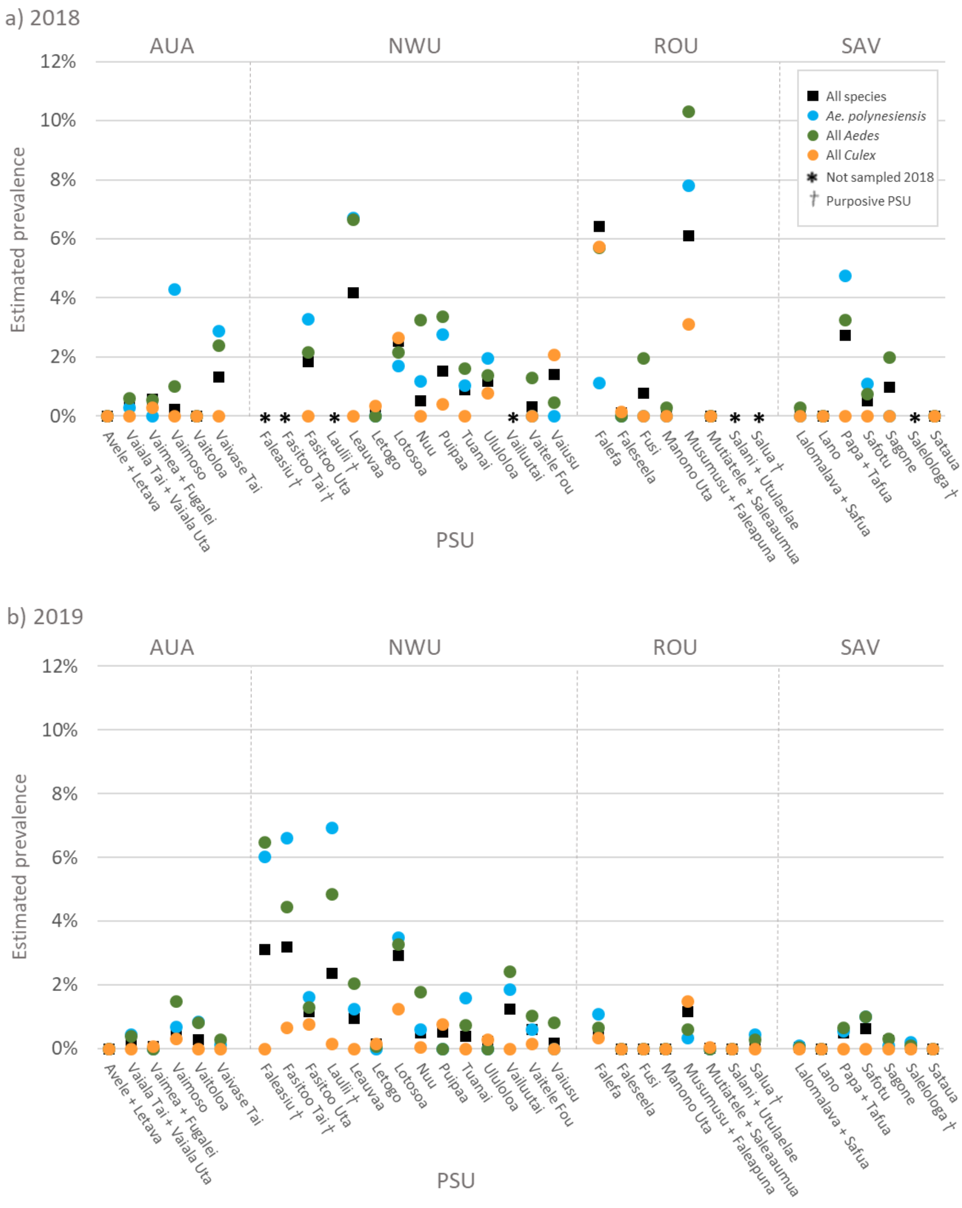

Figure S3. 1. Estimated prevalence of female mosquitoes infected with *W.bancrofti* by primary sampling unit (PSU) and species category in **a**) 2018 and **b**) 2019 in Samoa.

Table S3. 3. Estimated prevalence of female mosquitoes PCR-positive for *W. bancrofti* in 2018 by primary sampling unit (PSU) and species category in Samoa. (PSU = primary sampling unit, Prev = prevalence, CI = 95% confidence interval).

PSU	All Species			*Ae. polynesiensis*			All *Aedes*			All *Culex*		
	Prev	Lower CI	Upper CI	Prev	Lower CI	Upper CI	Prev	Lower CI	Upper CI	Prev	Lower CI	Upper CI
1	1.33%	0.40%	4.23%	2.88%	0.65%	13.10%	2.41%	0.73%	7.84%	0.00%	0.00%	2.26%
2	0.41%	0.09%	1.72%	0.29%	0.03%	2.80%	0.60%	0.13%	2.57%	0.00%	0.00%	1.57%
3	0.00%	0.00%	0.55%	0.00%	0.00%	1.26%	0.00%	0.00%	0.87%	0.00%	0.00%	1.49%
4	0.59%	0.13%	2.50%	0.00%	0.00%	17.48%	0.55%	0.05%	5.38%	0.30%	0.03%	2.81%
5	0.24%	0.02%	2.24%	4.29%	0.42%	36.71%	1.03%	0.10%	9.81%	0.00%	0.00%	1.19%
6	0.00%	0.00%	0.70%	0.00%	0.00%	3.49%	0.00%	0.00%	1.43%	0.00%	0.00%	1.35%
7	0.11%	0.01%	1.07%	0.00%	0.00%	0.77%	0.00%	0.00%	0.64%	0.35%	0.03%	3.39%
8	1.43%	0.43%	4.57%	0.00%	0.00%	5.20%	0.47%	0.04%	4.48%	2.08%	0.46%	9.39%
9	1.54%	0.46%	4.98%	2.77%	0.28%	39.07%	3.39%	0.75%	16.90%	0.42%	0.04%	4.00%
10	1.20%	0.43%	3.26%	1.97%	0.20%	21.74%	1.39%	0.30%	6.07%	0.80%	0.17%	3.43%
11	0.33%	0.03%	3.17%	0.00%	0.00%	19.22%	1.31%	0.13%	13.08%	0.00%	0.00%	1.73%
12	2.53%	1.42%	4.51%	1.70%	0.61%	4.65%	2.18%	0.96%	4.95%	2.65%	1.17%	6.13%
13	0.54%	0.16%	1.71%	1.20%	0.12%	13.67%	3.27%	1.00%	11.77%	0.00%	0.00%	0.50%
14	0.89%	0.32%	2.40%	1.06%	0.23%	4.56%	1.61%	0.58%	4.38%	0.00%	0.00%	1.10%
15	1.84%	0.86%	3.89%	3.29%	1.20%	9.47%	2.16%	1.01%	4.60%	0.00%	0.00%	3.63%
16	Not sampled in 2018											
17	4.18%	2.07%	8.67%	6.73%	3.01%	18.90%	6.66%	3.31%	14.82%	0.00%	0.00%	3.10%
18	Not sampled in 2018											
19	Not sampled in 2018											
20	Not sampled in 2018											
21	0.78%	0.17%	3.36%	0.00%	0.00%	16.02%	1.97%	0.44%	9.05%	0.00%	0.00%	1.67%
22	0.12%	0.01%	1.12%	0.00%	0.00%	4.58%	0.00%	0.00%	2.40%	0.15%	0.01%	1.39%
23	0.19%	0.02%	1.80%	0.00%	0.00%	4.81%	0.30%	0.03%	2.88%	0.00%	0.00%	1.98%
24	Not sampled in 2018											
25	0.00%	0.00%	0.62%	0.00%	0.00%	1.78%	0.00%	0.00%	0.86%	0.00%	0.00%	2.18%
26	6.43%	3.33%	13.67%	1.14%	0.11%	12.53%	5.71%	2.35%	16.00%	5.75%	2.16%	19.45%
27	6.10%	3.04%	13.46%	7.80%	2.48%	60.98%	10.32%	4.22%	53.36%	3.12%	0.96%	10.75%
	Not sampled in 2018											
29	0.12%	0.01%	1.09%	0.00%	0.00%	1.85%	0.29%	0.03%	2.72%	0.00%	0.00%	0.74%
30	0.00%	0.00%	0.67%	0.00%	0.00%	2.60%	0.00%	0.00%	1.39%	0.00%	0.00%	1.29%
31	0.53%	0.11%	2.26%	1.10%	0.24%	4.76%	0.77%	0.17%	3.27%	0.00%	0.00%	2.23%
32	0.00%	0.00%	1.28%	0.00%	0.00%	2.60%	0.00%	0.00%	1.81%	0.00%	0.00%	4.27%
33	0.99%	0.29%	3.14%	0.00%	0.00%	2.87%	2.00%	0.60%	6.50%	0.00%	0.00%	1.50%
34	2.74%	1.10%	6.70%	4.75%	1.73%	13.88%	3.26%	1.32%	8.06%	0.00%	0.00%	7.12%
35	Not sampled n 2018											

Table S3. 4. Estimated prevalence of female mosquitoes PCR-positive for *W. bancrofti* in 2019 by primary sampling unit (PSU) and species category in 2019 in Samoa. (PSU = primary sampling unit, Prev = prevalence, CI = 95% confidence interval).

PSU	All Species			*Ae. polynesiensis*			All *Aedes*			All *Culex*		
	Prev	Lower CI	Upper CI	Prev	Lower CI	Upper CI	Prev	Lower CI	Upper CI	Prev	Lower CI	Upper CI
1	0.18%	0.04%	0.74%	0.14%	0.01%	1.31%	0.29%	0.06%	1.21%	0.00%	0.00%	0.59%
2	0.26%	0.08%	0.83%	0.46%	0.14%	1.46%	0.41%	0.12%	1.29%	0.00%	0.00%	0.56%
3	0.00%	0.00%	0.24%	0.00%	0.00%	1.98%	0.00%	0.00%	1.00%	0.00%	0.00%	0.32%
4	0.07%	0.01%	0.62%	0.00%	0.00%	3.10%	0.00%	0.00%	1.01%	0.09%	0.01%	0.83%
5	0.58%	0.25%	1.29%	0.68%	0.06%	6.62%	1.50%	0.45%	4.75%	0.32%	0.09%	1.01%
6	0.30%	0.06%	1.27%	0.86%	0.08%	7.93%	0.84%	0.18%	3.49%	0.00%	0.00%	0.61%
7	0.15%	0.04%	0.48%	0.00%	0.00%	0.41%	0.08%	0.01%	0.76%	0.15%	0.03%	0.61%
8	0.17%	0.02%	1.60%	0.00%	0.00%	8.36%	0.83%	0.08%	7.52%	0.00%	0.00%	0.83%
9	0.54%	0.11%	2.26%	0.00%	0.00%	3.77%	0.00%	0.00%	2.23%	0.77%	0.16%	3.27%
10	0.25%	0.07%	0.77%	0.00%	0.00%	2.29%	0.00%	0.00%	1.15%	0.29%	0.09%	0.93%
11	0.61%	0.22%	1.64%	0.60%	0.06%	5.57%	1.03%	0.31%	3.25%	0.15%	0.01%	1.41%
12	2.94%	2.07%	4.20%	3.50%	2.34%	5.29%	3.27%	2.25%	4.77%	1.26%	0.38%	4.04%
13	0.49%	0.23%	1.03%	0.61%	0.06%	5.71%	1.78%	0.77%	3.96%	0.05%	0.00%	0.46%
14	0.38%	0.11%	1.21%	1.59%	0.48%	5.03%	0.75%	0.22%	2.35%	0.00%	0.00%	0.61%
15	1.17%	0.47%	2.82%	1.62%	0.35%	7.05%	1.31%	0.39%	4.14%	0.76%	0.16%	3.24%
16	1.26%	0.55%	2.80%	1.87%	0.56%	6.12%	2.43%	1.07%	5.46%	0.00%	0.00%	0.91%
17	0.95%	0.38%	2.29%	1.26%	0.27%	5.33%	2.04%	0.82%	4.90%	0.00%	0.00%	0.77%
18	3.19%	2.07%	4.93%	6.63%	4.03%	11.51%	4.46%	2.83%	7.11%	0.67%	0.14%	2.84%
19	3.12%	2.05%	4.76%	6.04%	3.66%	10.40%	6.50%	4.25%	10.16%	0.00%	0.00%	0.58%
20	2.38%	1.40%	4.01%	6.94%	3.81%	13.30%	4.87%	2.80%	8.51%	0.16%	0.02%	1.54%
21	0.00%	0.00%	0.18%	0.00%	0.00%	1.64%	0.00%	0.00%	0.67%	0.00%	0.00%	0.25%
22	0.00%	0.00%	0.11%	0.00%	0.00%	2.75%	0.00%	0.00%	1.22%	0.00%	0.00%	0.12%
23	0.00%	0.00%	0.20%	0.00%	0.00%	3.05%	0.00%	0.00%	1.22%	0.00%	0.00%	0.24%
24	0.00%	0.00%	0.05%	0.00%	0.00%	0.18%	0.00%	0.00%	0.13%	0.00%	0.00%	0.09%
25	0.02%	0.00%	0.23%	0.00%	0.00%	0.28%	0.00%	0.00%	0.17%	0.05%	0.01%	0.51%
26	0.53%	0.19%	1.42%	1.09%	0.24%	4.62%	0.68%	0.14%	2.84%	0.34%	0.07%	1.44%
27	1.16%	0.50%	2.58%	0.34%	0.03%	3.22%	0.62%	0.13%	2.61%	1.50%	0.53%	4.03%
28	0.26%	0.05%	1.08%	0.44%	0.09%	1.87%	0.30%	0.06%	1.27%	0.00%	0.00%	2.23%
29	0.04%	0.00%	0.37%	0.09%	0.01%	0.85%	0.05%	0.00%	0.45%	0.00%	0.00%	0.87%
30	0.00%	0.00%	0.23%	0.00%	0.00%	0.58%	0.00%	0.00%	0.31%	0.00%	0.00%	0.92%
31	0.65%	0.26%	1.56%	1.01%	0.36%	2.72%	1.02%	0.41%	2.46%	0.00%	0.00%	0.76%
32	0.00%	0.00%	0.24%	0.00%	0.00%	0.50%	0.00%	0.00%	0.38%	0.00%	0.00%	0.66%
33	0.27%	0.08%	0.85%	0.11%	0.01%	0.99%	0.33%	0.10%	1.03%	0.00%	0.00%	1.19%
34	0.51%	0.25%	1.01%	0.56%	0.22%	1.35%	0.66%	0.32%	1.33%	0.00%	0.00%	0.55%
35	0.06%	0.01%	0.54%	0.22%	0.02%	2.07%	0.11%	0.01%	0.99%	0.00%	0.00%	0.50%

4. Participant numbers for human survey

Table S4. 1. Number of participants in each of the four regions of Samoa in the 28 randomly selected primary sampling units included in 2018 and 2019 human and mosquito surveys. AUA = Apia Urban Area; NWU = North West Upolu; ROU = Rest of Upolu; SAV = Savai'i.

Region	2018		2019	
	5-9 year-olds	≥ 10 year-olds	5-9 year-olds	≥ 10 year-olds
AUA	299	349	339	323
NWU	572	567	596	699
ROU	340	342	393	394
SAV	331	293	368	371

Table S4. 2. Number of participants in each of the four regions of Samoa in the 30 randomly selected primary sampling units included in 2018 and 2019 human surveys. AUA = Apia Urban Area; NWU = North West Upolu; ROU = Rest of Upolu; SAV = Savai'i.

Region	2018		2019	
	5-9 year-olds	≥ 10 year-olds	5-9 year-olds	≥ 10 year-olds
AUA	299	349	339	323
NWU	640	621	648	665
ROU	398	402	456	456
SAV	331	293	368	371

5. Distribution of PCR-positive mosquitoes and Ag-positive humans

The spatial distribution of MX and human Ag results. PCR-positive mosquitoes and Ag-positive humans were found in all regions in both years. In 2018, of the 23 PSUs with PCR-positive pools, nine (39%) did not return any positive *Ae. polynesiensis* pools; in 2019, this proportion reduced to 21% (six out of 28 PSUs).

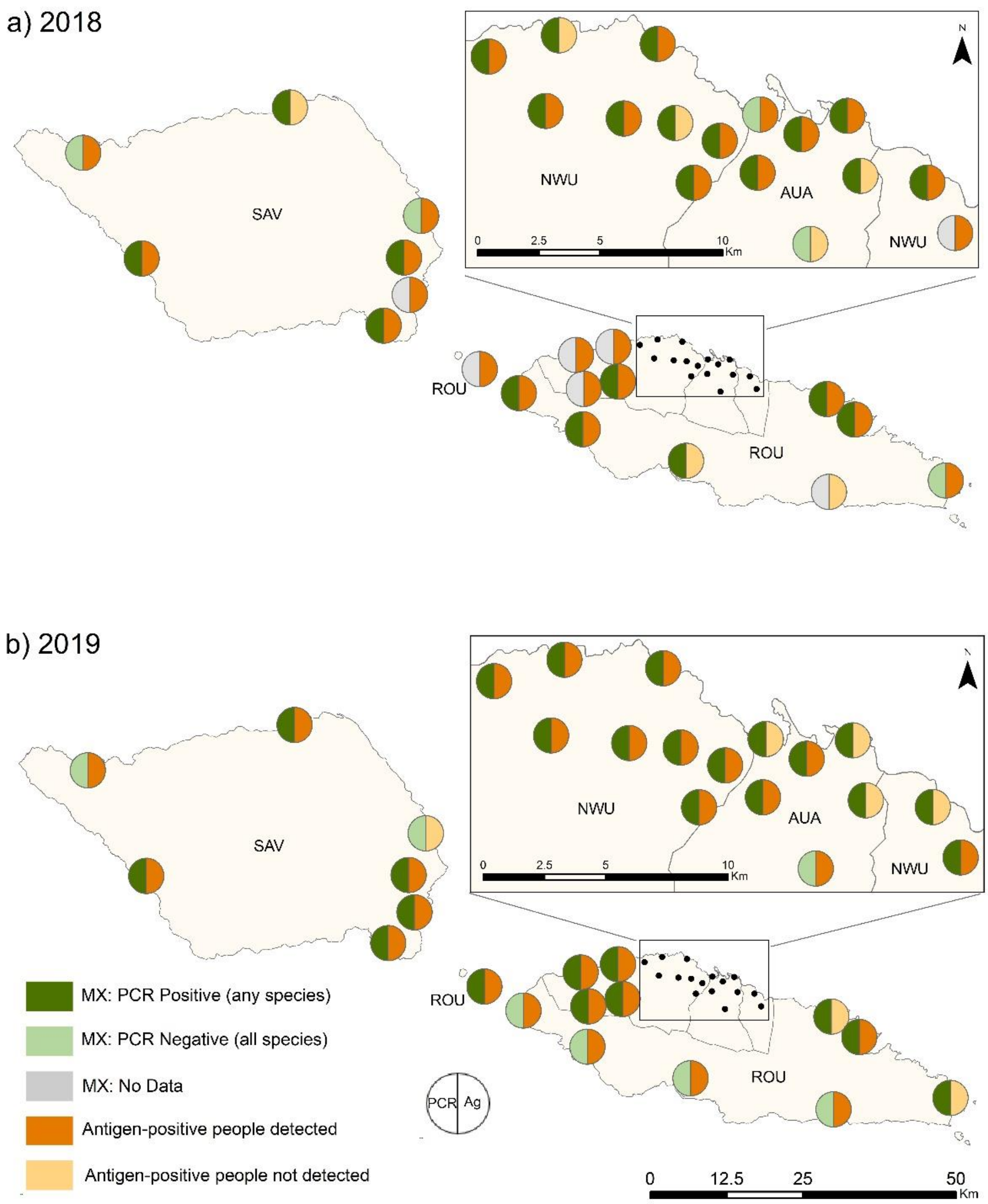

Figure S5. 1 Distribution of PCR-positive mosquitoes and Ag-positive humans by primary sampling unit in **a**) 2018 and **b**) 2019 in Samoa. Molecular Xenomonitoring (MX) results for 'any species' shown in the left hemisphere and human Ag results shown in the right hemisphere.

6. Change in prevalence of PCR-positive mosquitoes from 2018 to 2019

Table S6. 1. Odds ratios for change in prevalence of female mosquitoes PCR-positive for *W. bancrofti*, between 2018 and 2019 in the 28 common PSUs, adjusted for species. AUA=Apia Urban Area; NWU=North West Upolu; ROU=Rest of Upolu; SAV=Savai'i.

PSU	PSU Name	Region	OR* 2019 vs 2018	95% CrI
1	Vaivase Tai	AUA	0.30	0.09-0.78
2	Vaiala Tai + Vaiala Uta	AUA	0.42	0.17-1.16
3	Avele + Letava	AUA	0.39	0.09-1.81
4	Vaimea + Fugalei	AUA	0.34	0.10-0.99
5	Vaimoso	AUA	0.54	0.23-1.76
6	Vaitoloa	AUA	0.53	0.19-2.05
7	Letego	NWU	0.46	0.17-1.40
8	Vaiusu	NWU	0.36	0.12-1.01
9	Puipaa	NWU	0.37	0.13-1.00
10	Ululoloa	NWU	0.35	0.13-0.85
11	Vaitele Fou	NWU	0.46	0.18-1.50
12	Lotosoa	NWU	0.65	0.35-1.31
13	Nuu	NWU	0.46	0.21-1.19
14	Tuanai	NWU	0.40	0.16-0.96
15	Fasitoo Uta	NWU	0.52	0.24-1.35
17	Leauvaa	NWU	0.32	0.13-0.67
21	Fusi	ROU	0.27	0.06-0.79
22	Faleseela	ROU	0.31	0.08-1.08
23	Manono Uta	ROU	0.33	0.08-1.13
25	Mutiatele + Saleaaumua	ROU	0.39	0.10-1.57
26	Falefa	ROU	0.22	0.07-0.52
27	Musumusu + Faleapuna	ROU	0.26	0.09-0.59
29	Lalomalava + Safua	SAV	0.36	0.10-1.15
30	Lano	SAV	0.37	0.09-1.50
31	Safotu	SAV	0.50	0.22-1.36
32	Sataua	SAV	0.36	0.08-1.51
33	Sagone	SAV	0.33	0.12-0.81
34	Papa + Tafua	SAV	0.31	0.13-0.65

* odds ratios closely approximate prevalence ratios since prevalence of LF DNA was low in all vector species, all PSUs, and both years. ORs <1 indicate decrease in infection prevalence in 2019 compared to 2018, ORs >1 indicate an increase, and OR of 1 indicate no change.

7. Sensitivity Analysis for change in prevalence of PCR-positive mosquitoes between 2018 and 2019

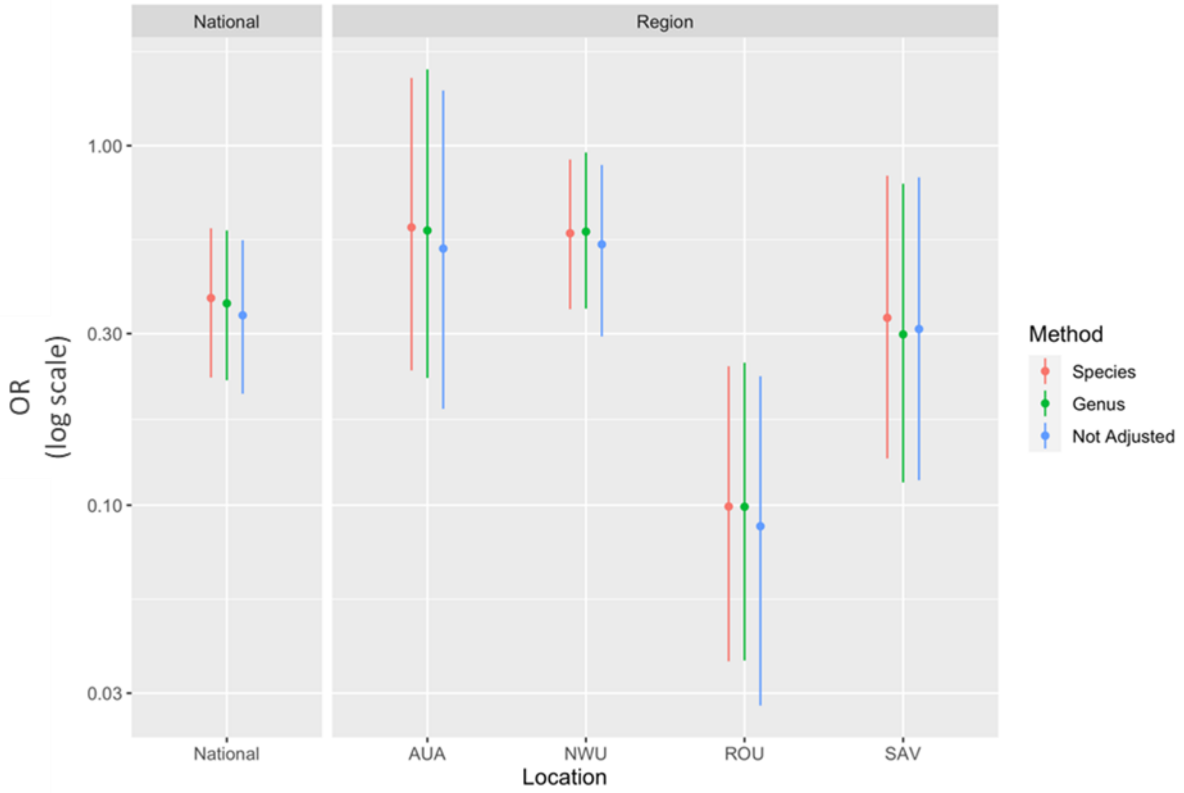

Figure S7. 1. Change in-mosquito infection prevalence by region from 2018 to 2019, expressed as an odds ratio (OR), using different methods of adjusting for mosquito categories: Species (2018 categorisation), Genus (i.e. *Aedes* vs *Culex*) or no adjustment. Given the low prevalence across measures, the ORs are approximately equal to prevalence ratios. ORs <1 indicate decrease in infection prevalence in 2019 compared to 2018, ORs >1 indicate an increase, and OR of 1 indicate no change. Note log scale on Y Axis. AUA = Apia Urban Area; NWU = North West Upolu; ROU = Rest of Upolu; SAV = Savai'i.

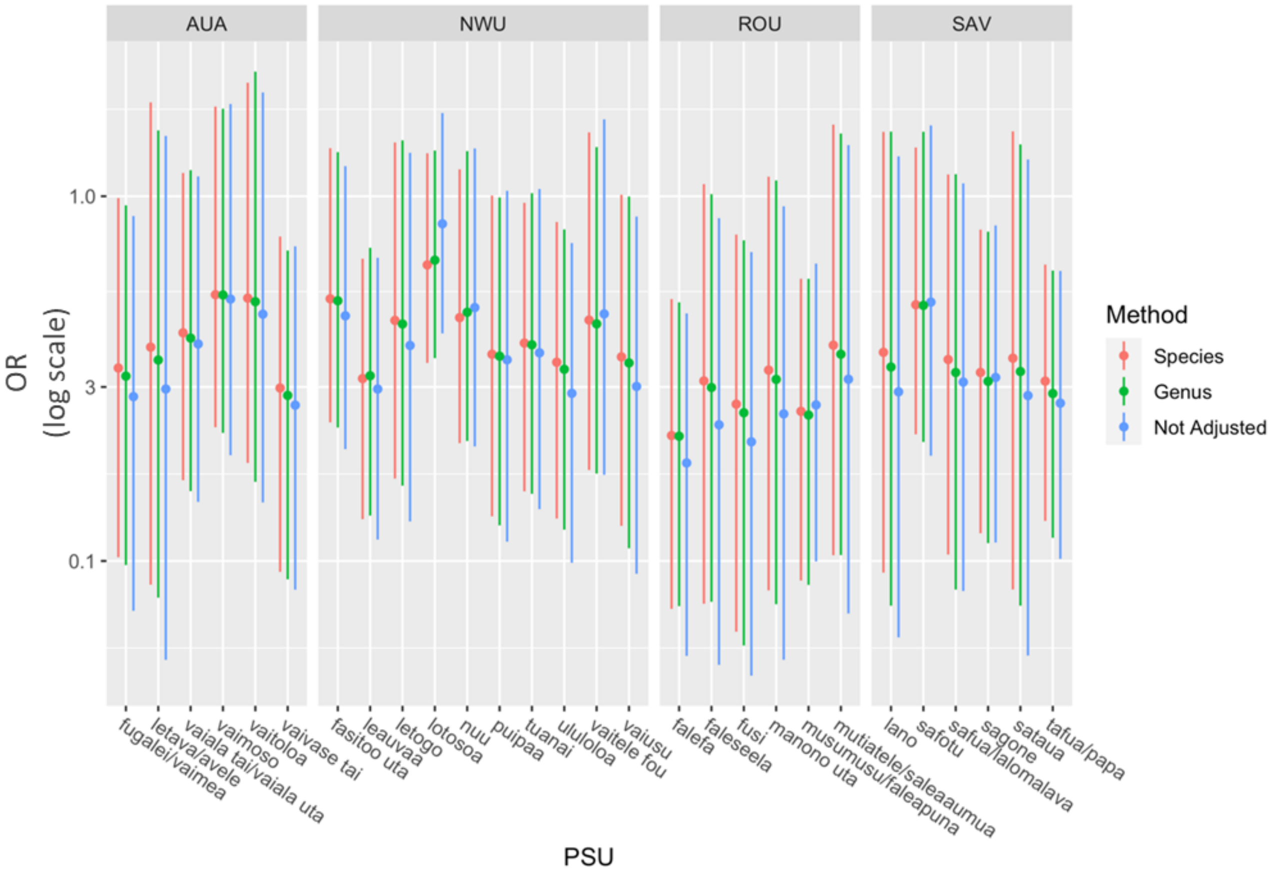

Figure S7. 2. Change in mosquito infection prevalence by primary sampling unit (PSU) from 2018 to 2019, expressed as an odds ratio (OR), for mosquito infection prevalence using different methods of adjusting for mosquito categories: Species (2018 categorisation), Genus *Aedes* vs *Culex* or no adjustment. Given the low prevalence across measures, the ORs are approximately equal to prevalence ratios. ORs <1 indicate decrease in infection prevalence in 2019 compared to 2018, ORs >1 indicate an increase, and OR of 1 indicate no change. Note log scale on Y Axis. AUA = Apia Urban Area; NWU = North West Upolu; ROU = Rest of Upolu; SAV=Savai'i.
